# Identification of Novel microRNAs in Post-Transcriptional Control of Nrf2 Expression and Redox Homeostasis in Neuronal, SH-SY5Y Cells

**DOI:** 10.1371/journal.pone.0051111

**Published:** 2012-12-07

**Authors:** Madhusudhanan Narasimhan, Dhyanesh Patel, Dhanashree Vedpathak, Marylatha Rathinam, George Henderson, Lenin Mahimainathan

**Affiliations:** 1 Department of Pharmacology and Neuroscience, Texas Tech University Health Sciences Center, Lubbock, Texas, United States of America; 2 South Plains Alcohol and Addiction Research Center, Texas Tech University Health Sciences Center, Lubbock, Texas, United States of America; North Carolina State University, United States of America

## Abstract

Nuclear factor-erythroid 2-related factor 2 (Nrf2/NFE2L2), a redox-sensitive transcription factor plays a critical role in adaptation to cellular stress and affords cellular defense by initiating transcription of antioxidative and detoxification genes. While a protein can be regulated at multiple levels, control of Nrf2 has been largely studied at post-translational regulation points by Keap1. Importantly, post-transcriptional/translational based regulation of Nrf2 is less understood and to date there are no reports on such mechanisms in neuronal systems. In this context, studies involving the role of microRNAs (miRs) which are normally considered as fine tuning regulators of protein production through translation repression and/or post-transcriptional alterations, are in place. In the current study, based on *in-silico* analysis followed by immunoblotting and real time analysis, we have identified and validated for the first time that human NFE2L2 could be targeted by miR153/miR27a/miR142-5p/miR144 in neuronal, SH-SY5Y cells. Co-transfection studies with individual miR mimics along with either WT 3′ UTR of human Nrf2 or mutated miRNA targeting seed sequence within Nrf2 3′ UTR, demonstrated that Nrf2 is a direct regulatory target of these miRs. In addition, ectopic expression of miR153/miR27a/miR142-5p/miR144 affected Nrf2 mRNA abundance and nucleo-cytoplasmic concentration of Nrf2 in a Keap1 independent manner resulting in inefficient transactivating ability of Nrf2. Furthermore, forced expression of miRs diminished GCLC and GSR expression resulting in alteration of Nrf2 dependent redox homeostasis. Finally, bioinformatics based miRNA-disease network analysis (MDN) along with extended computational network analysis of Nrf2 associated pathologic processes suggests that if in a particular cellular scenario where any of these miR153/miR27a/miR142-5p/miR144 either individually or as a group is altered, it could affect Nrf2 thus triggering and/or determining the fate of wide range of disease outcomes.

## Introduction

NF-E2 related factor 2 (NFE2L2/Nrf2), a redox sensitive transcription factor responds to oxidative insult by regulating a battery of cytoprotective genes including those involved in glutathione metabolism [1]–[3]. Basal homeostatic levels of Nrf2 in any cellular system are predominantly maintained by quenching the interaction of Nrf2 in cytosol with Keap1, a Cullin 3-dependent substrate adaptor protein [4]. Upon exposure to electrophilic stress stimuli, Nrf2 dissociates from Keap1 thus eluding proteasomal degradation. It is shuttled to nucleus where it binds to critical cis-acting antioxidant response element (ARE) and triggers the transactivation of its targets [1], [5]. Nrf2 is considered as one of the chief ARE binding transactivators [5] and since its discovery as a protein controlling the expression of â-globin gene [6], numerous reports have documented beneficial role of Nrf2 in affording antioxidant cytoprotection in various disease settings [7]. Both clinical and experimental evidences (cell and animal models) have demonstrated a strong correlation between dysregulation in Nrf2 pathway and various neurological diseases [8]–[11]. In addition to widely studied Keap1 based Nrf2 control, there have been reports addressing transcriptional, translational and phosphorylation based posttranslational control of Nrf2 expression [2], [12]–[14]. Yet, the mechanisms elucidating the posttranscriptional control of Nrf2 are scant.

Recently, a novel class of posttranscriptional regulators, microRNAs (miRs) which are short non-coding RNAs of ∼21–23 nucleotides in length have been shown to effect translation repression or degradation of a target mRNA or both in a sequence-specific manner [15]. Of great interest in emerging field of miRs, it is to be noted that miravirsen (antagomiR for 122) have been successfully tested in Phase 2a trials for treating naïve patients with chronic HCV genotype 1 infection [16]. Research exploring the importance of miRs in brain gene regulation in health and disease has gained considerable momentum as is evident from the reports in postmortem brain samples of most common neurodegenerative disorders such as Alzheimer’s (AD), Parkinson’s disease (PD), amyotrophic lateral sclerosis (ALS), Huntington’s disease (HD), Frontotemporal lobar degeneration (FTLD) [17]–[21]. So far, there are only few studies in non-neuronal models that has validated Nrf2 silencing by miR144 [22], miR28 [23] and miR34a [24].

**Table 1:** Sequences of mutagenic oligonucleotides.

miRNA site mutated in human Nrf2 3′ UTR	Primers
miR153	For: 5′– AAGTAATTCTAaagAAATCATAGCCAAAACTAGTA –3′Rev: 5′ – TATGATTTcttTAGAATTACTTATAAAGTATGA –3′
miR27a	For: 5′ – GCTCCTACacaGATGTGAAATGCTCATACTT –3′Rev: 5′ – TTTCACATCtgtGTAGGAGCTTTTAGTATAATAGTA –3′
miR142-5p	For: 5′ – TGCTCATACacaATAAGTAATTCTATGCAAAATCATA –3′Rev: 5′ – AATTACTTATtgtGTATGAGCATTTCACATCA –3′

**Figure 1:**
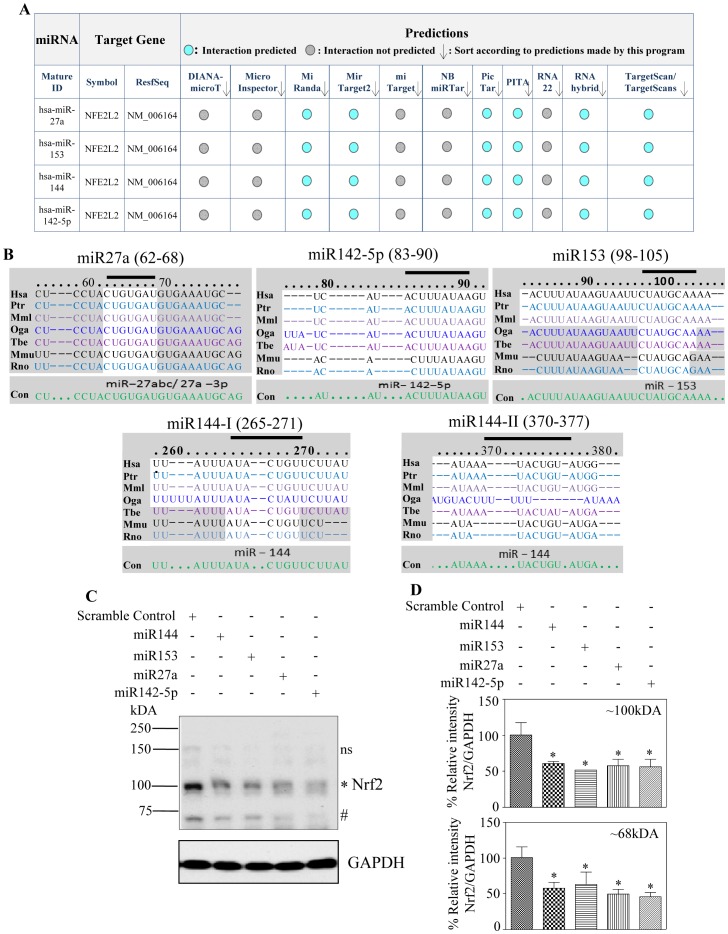
Overexpression of miR144, miR153, miR27a and miR142-5p downregulates Nrf2 protein expression in SH-SY5Y cells. (A) *In-silico* bioinformatic algorithm based prediction using “miRecords” to identify different miRs that target human Nrf2 3′ UTR and the top 4 miRs generated by atleast 6 individual databases are listed. (B) Sequence alignment and comparison of evolutionary conservation between different miRNA (miR27a, miR142-5p, miR153, miR144(site1), miR144(site2)) seed sequences and its canonical targeting sequences on human Nrf2 3′-UTRs in cross species. Hsa, human; Ptr, chimpanzee; Mml, rhesus; Oga, bushbaby; Tbe, treeshrew; Mmu, mouse; Rno, rat. The sequences recognized by individual miRNA on human Nrf2 3′ UTR are marked above with a black, bold line. (C) SH-SY5Y cells were transiently transfected with 100 nM each of indicated miR mimics individually for 48 h. Whole cell protein lysates were immunoblotted for Nrf2. GAPDH and tubulin were used for normalization and a representative immunoblot is shown. (D) * and # indicate 100 kDA and 68 kDA Nrf2 bands respectively and ns indicate non-specific bands. Using ImageJ based densitometric analysis, the intensity of 100 kDA and 68 kDA Nrf2 levels were normalized to GAPDH and plotted. Comparison between scramble control miR and indicated miRs were performed using one way ANOVA followed by Newman-Keul’s post-test analysis. Values are means±s.e.m from 4 independent replicates and * indicates significantly different from scramble control miR (p<0.05).

Given the strong causal relationship between oxidative stress and various pathologies of CNS, it is of paramount importance to thoroughly understand the complex regulation of “master redox switch”, Nrf2 in neuronal models. Thus, identification of miR based dysregulation of Nrf2 in neuronal models will generate an understanding of how neuron-based cytoprotection machinery can be perturbed in pathologic settings and enable development of potential Nrf2-dependent neuroprotection strategies. In this context, our current study has demonstrated a model of posttranscriptional repression of Nrf2 and its associated redox homeostasis by novel miRNAs 153/27a/142-5p/144 in a SH-SY5Y neuronal cellular model. However, future studies will need to determine the importance of association of Nrf2 deficiency with respect to these miRs and its implication in oxidative stress dependent neurodegeneration.

**Figure 2:**
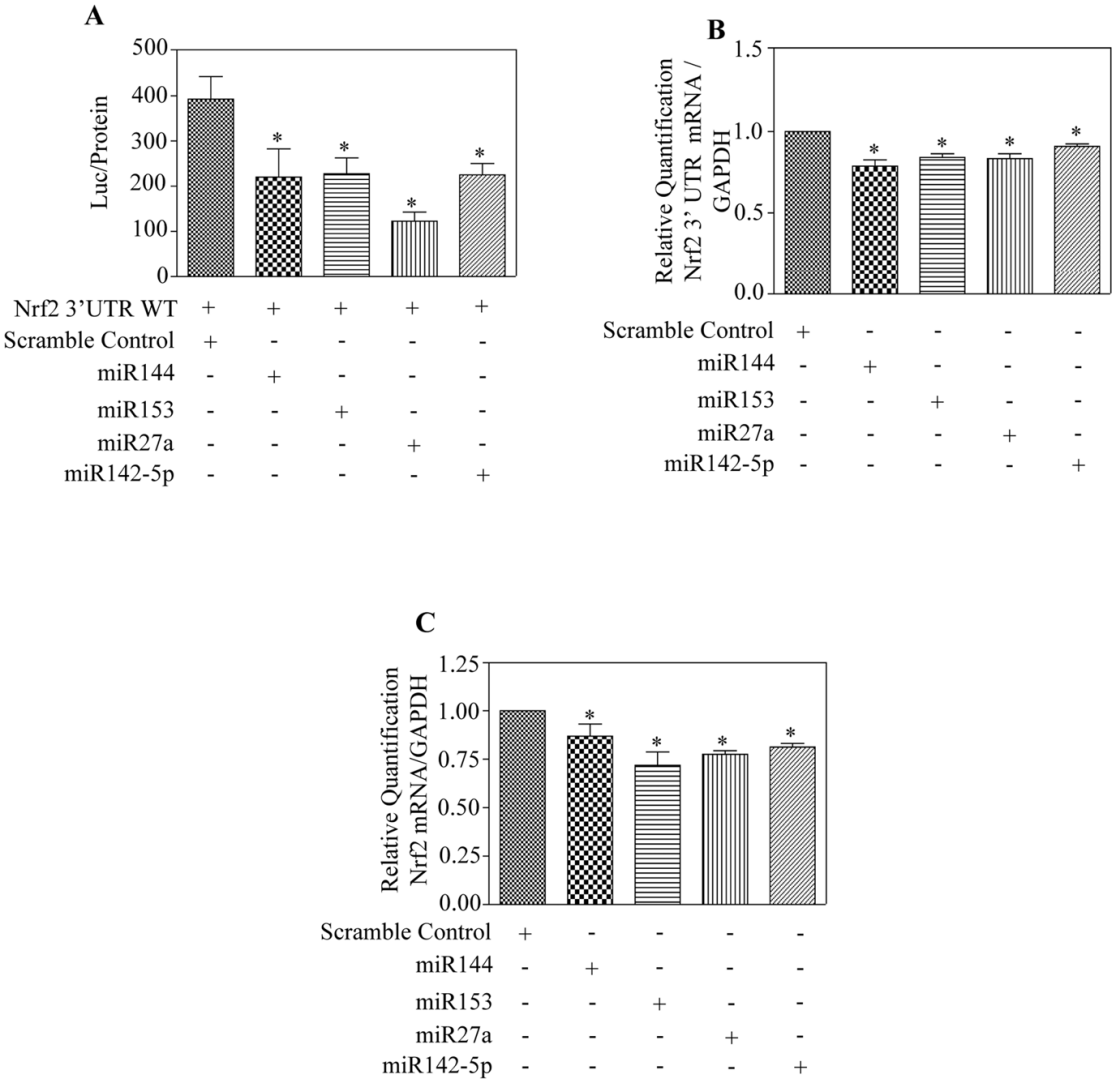
Individual overexpression of miR144, miR153, miR27a and miR142-5p directly target Nrf2 3′ UTR and downregulate expression of Nrf2 transcript. (A) SH-SY5Y cells were co-transfected with 100 nM of indicated miRs along with wildtype Nrf2 3′UTR construct as described in “Materials and Methods”. After 48 h, luciferase reporter assay was performed and the relative luciferase normalized to protein was graphed (n = 4). (B) Cells were transfected with different miRs for 48 h and Taqman based real time qRT-PCR analysis was performed using primers specific against Nrf2 3′ UTR and GAPDH. Endogenous expression of Nrf2 3′ UTR was quantified relative to GAPDH levels from 4 independent samples. (C) TaqMan based quantitative real time RT-PCR analysis of Nrf2 mRNA level in SH-SY5Y cells transiently transfected with indicated miRs. GAPDH mRNA levels were used to normalize those of Nrf2 mRNA (n = 5). In panel (A–C), one way ANOVA was used to establish the statistical significance and * - p<0.05 compared with scramble miR control.

## Materials and Methods

### Materials

SH-SY5Y neuroblastoma cells (CRL-2266) was purchased from ATCC (Manassas, VA). Eagle’s minimum essential medium (MEM), antibiotic/antimycotic solution were from Invitrogen (Carlsbad, CA). Nutrient mixture F-12 Ham was obtained from Sigma-Aldrich (St. Louis, MO). Fetal Bovine Serum was from Atlanta biologicals (Lawrenceville, GA) and plasmocin from Invivogen (San Diego, CA). miRNA precursors for hsa-miR144, hsa-miR153, hsa-miR27a, hsa-miR142-5p, hsa-miR21, scramble control miR, siPort™ Amine NeoFX and mirVana miRNA isolation kit were purchased from Ambion (Austin, TX). FuGENE HD was from Roche Diagnostics (Indianapolis, IN). QuantiTect reverse transcription kit for first-strand synthesis was purchased from QIAGEN (Valencia, CA). Antibodies for Nrf2, PTEN, GCLC, GSR, tubulin, GAPDH, lamin b1 and HRP-conjugated goat secondary antibody were from SantaCruz Biotechnologies Inc. (Santa Cruz, CA). HRP conjugated rabbit secondary antibody was bought from Jackson ImmunoResearch Laboratories Inc. (West Grove, PA). NQO1 ARE Luc plasmid from Dr. Roland C. Wolf (Dundee, UK) and Nrf2 3′ UTR WT and Nrf2 3′ UTR mut 144 constructs from Dr. Jen-Tsan Chi (Durham, USA) were kind gift. PureYield Plasmid miniprep kit and GSH/GSSG-Glo Assay kit was obtained from Promega (Madison, WI). NEB10-beta competent E.coli cells and DpnI restriction enzyme were purchased from NewEngland Biolabs, (Ipswich, MA). PrimeSTAR Max DNA polymerase was a kind gift for trial from Takara Bio USA Inc. (Mountain View, CA). 2′,7′-dichlorodihydrofluorescein diacetate (DCF-DA) was obtained from Calbiochem (La Jolla, CA). Antibody for actin and all other reagents were from Sigma-Aldrich (St. Louis, MO).

**Figure 3:**
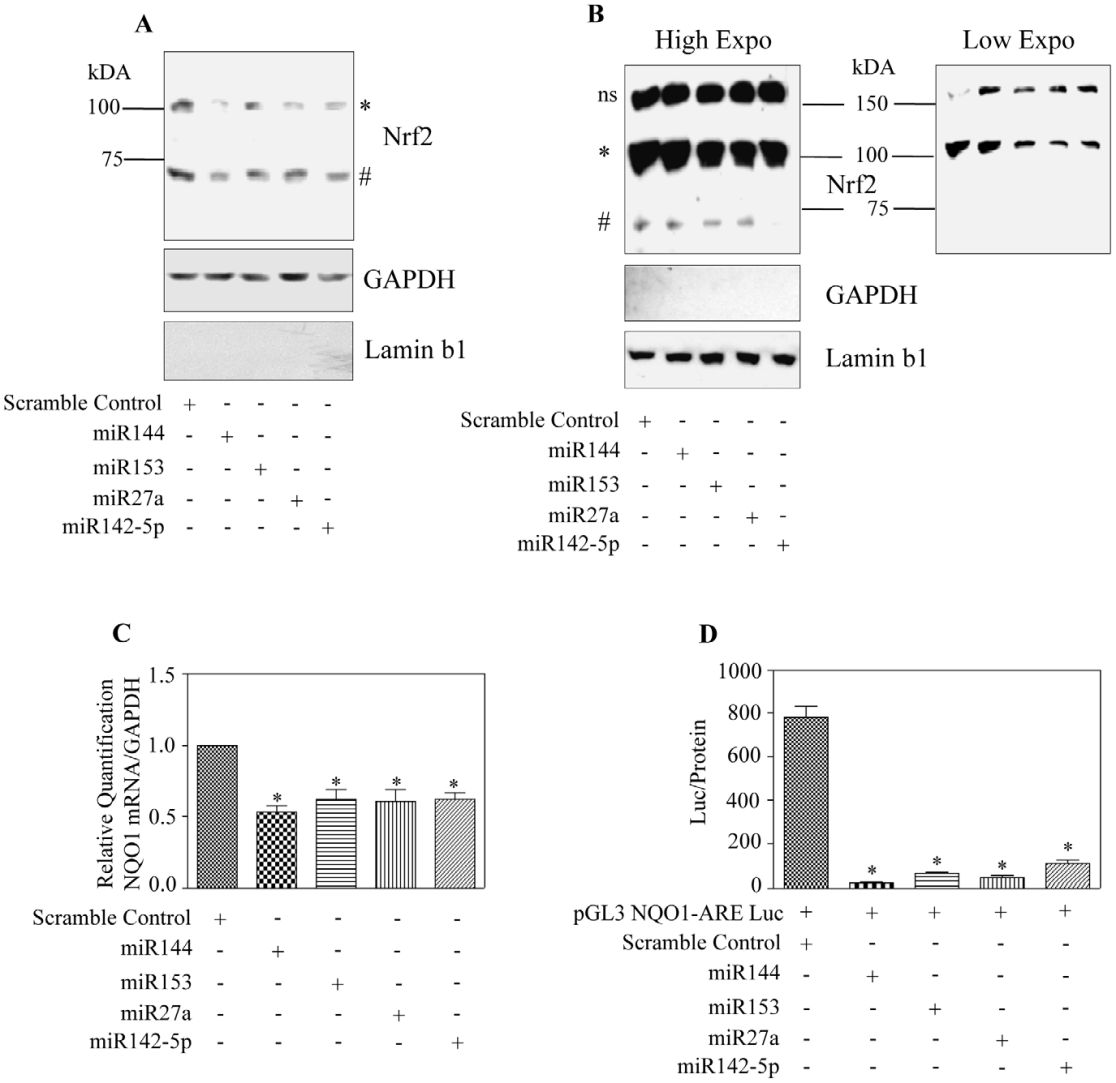
Nrf2 transactivation and ARE-driven NQO1 gene expression were reduced by overexpression of different miRs, miR144, miR153, miR27a and miR142-5p. (A) Cytosolic and (B) nuclear extracts from either scramble miR or indicated miR transfected cells were immunoblotted for Nrf2. GAPDH and laminb1 served as controls for purity of cytosolic and nuclear fraction respectively. * and # indicate 100 kDA and 68 kDA Nrf2 bands respectively and ns indicate non-specific bands. Low exposure of Panel B was provided to appreciate the reduction in 100 kDA. Three independent experiments were performed and a representative blot is given in A & B. (C) Endogenous NQO1 mRNA expression from cells transfected with or without individual miRs were estimated by Taqman based real-time qRT-PCR analysis. NQO1 mRNA levels were normalized to GAPDH (n = 5). (D) As indicated, 48 h after co-transfection of luciferase constructs containing NQO1 ARE sequences with individual miRs, Nrf2 transactivation was determined in terms of measuring the reporter activity. The graph represents the luciferase activity normalized to protein values (n = 6). Panel (C & D), * indicates significant differences between miR and scramble control miR transfected cells as analyzed by one way ANOVA followed by Newman Keul’s post-test.

### Cell Culture

SH-SY5Y cells were sub-cultured and maintained at 37°C and 5% CO2 atmospheric condition in 1∶1 minimum essential medium and F-12 HAM nutrient mixture supplemented with 10% FBS, antibiotic/antimycotic and plasmocin. All experiments were performed at cell confluency of 70% to 80% between passages 26–31.

**Figure 4:**
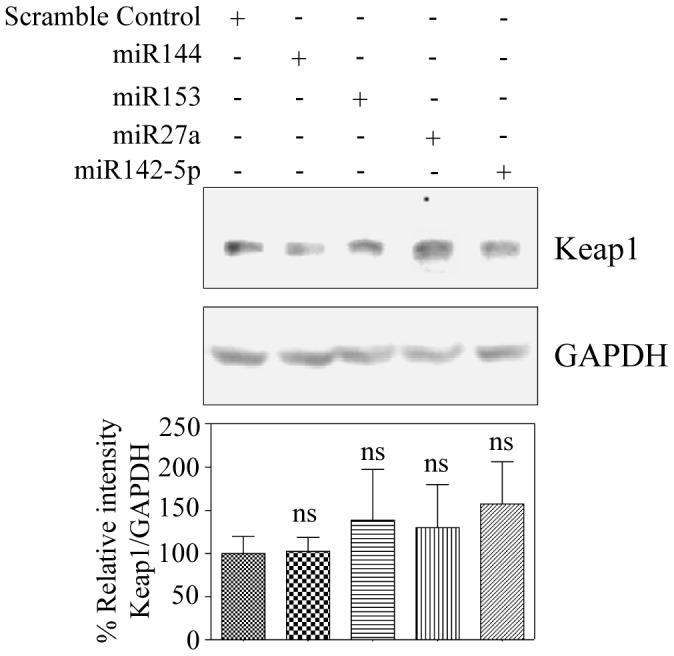
miRs144/153/27a/142-5p repression of Nrf2 is Keap1 independent. (A) Indicated miR mimics or scramble miR mimic was transfected into SH-SY5Y cells for 48 h. 30 µg of whole cell proteins from different samples was immunoblotted for Keap1. GAPDH was used as loading control. Lower panel depicts densitometric analysis of Keap1 normalized to GAPDH (n = 5). ns indicates not significant (p<0.05) vs scramble control miR transfected cells as analyzed by one way ANOVA followed by Newman Keul’s post-test.

### Transient Transfection

SH-SY5Y cells were transiently transfected with 100 nM of indicated pre-miRs and 200 ng of plasmid reporter constructs. For the experiments involving transfection of miR alone, siPort amine was used as transfection agent and Fugene HD was used in the experiments involving co-transfection of plasmid constructs and miRNA. Briefly, miRs (or) vector constructs and respective transfection reagents were appropriately diluted in OPTI-MEM I medium separately and incubated for 5 min. The mixed reagents were incubated at room temperature for 20 min allowing the formation of transfection complex. Transfection was performed in serum free, antibiotic free media and 1.5 h post-transfection media containing serum, antibiotics was added and the plates were returned to incubator. After 48 h, cells were processed for various downstream applications.

**Figure 5:**
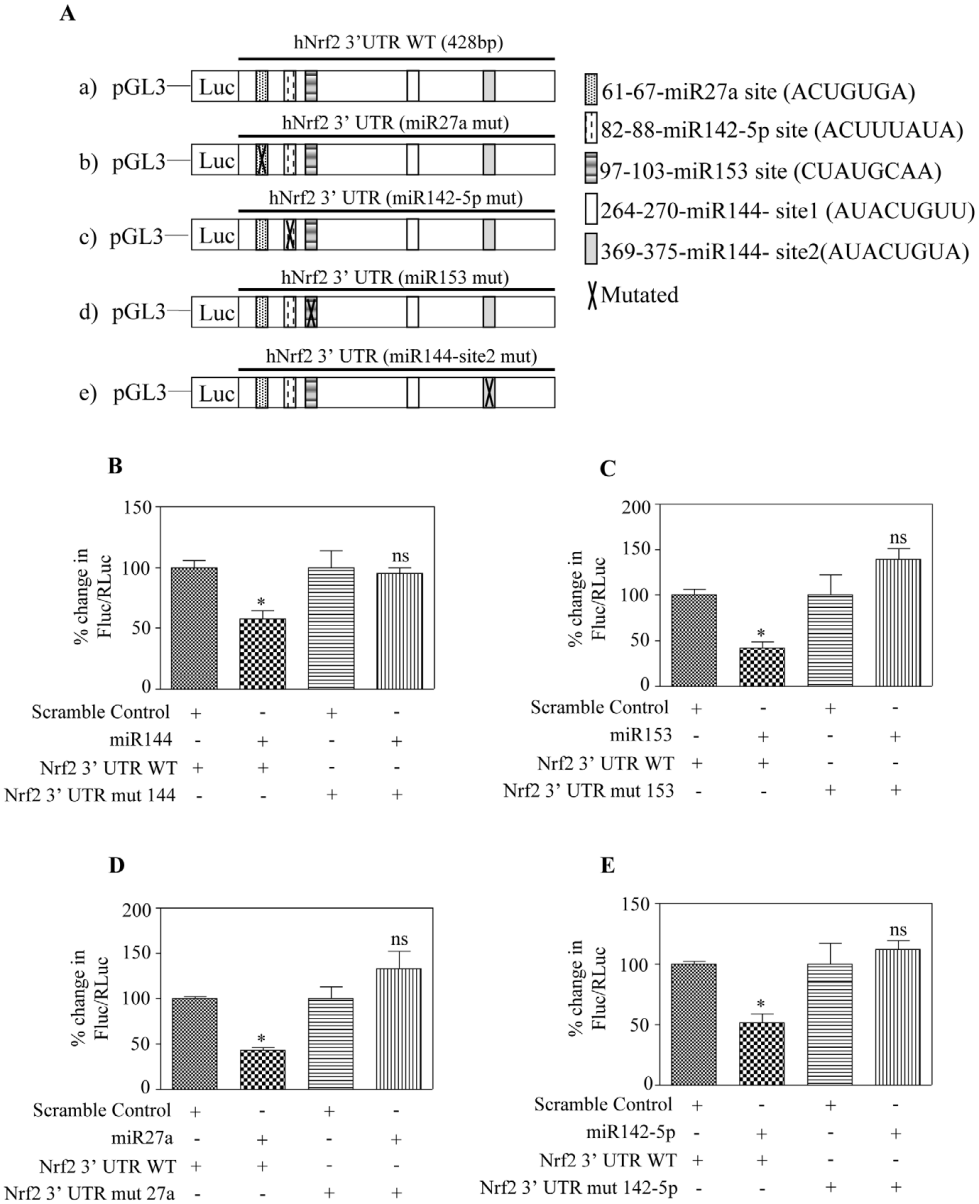
Mutating miR144, miR153, miR27a and miR142-5p binding sites in Nrf2 3′ UTR confirms Nrf2 as a direct target of miR144/153/27a/142-5p. (A) Schematic representation of different Nrf2 3′ UTR luciferase reporters used in the transfection experiments is depicted. Constructs a, and e were generated in Dr. Jen-Tsan Chi’s laboratory and constructs b, c, d were generated in our laboratory and sequence verified for incorporation of mutagenized nucleotides (data not shown). (B) SH-SY5Y cells were co-transfected with either miR144 or scramble miR and 200 ng pGL3 reporter construct containing wild type or miR144 site mutated 3′ UTR of Nrf2. The relative firefly luciferase activity normalized with renilla luciferase was measured 48 h post-transfection and results are plotted as percentage change over respective control. (C) Cells were co-transfected with both the reporter gene (either wild type or miR 153 site mutated Nrf2 3′ UTR) constructs and scramble miR mimic/miR153 mimic. 48 h following transfection, firefly luciferase activity normalized to renilla activity was determined. The results are represented as percentage change over respective controls. (D) 100 nM of pre-miR scramble control (or) pre-miR27a was transfected into SH-SY5Y cells together with a wild-type or miR27a binding site mutated Nrf2 3′ UTR construct. Cells were lysed 48 h post-transfection and luciferase signal was measured. Firefly luciferase signals were normalized to renilla luciferase signal and percentage change over corresponding controls was represented. (E) Luciferase reporters containing wild-type or miR142-5p site mutant of human Nrf2 3′ UTR were co-transfected with scramble control miRNA or miR142-5p precursors into SH-SY5Y cells. 48 h after transfection, dual luciferase activity was measured. After normalization for renilla luciferase activity, the results were plotted as percentage change and compared to the corresponding controls. In (B-E), values are expressed as means±s.e.m from 3 independent samples and * indicate significantly (p<0.05) different when compared with wild-type Nrf2 3′ UTR construct by one way ANOVA/Newman Keuls post-test; ns-not significant when compared with the respective miRNA target site mutated Nrf2 3′ UTR constructs.

### Immunoblotting

Following experimental treatments, SH-SY5Y cells were gently washed with cold PBS and lysed in ice-cold RIPA lysis buffer. The lysates were sonicated and supernatants were collected by centrifuging at 13,000 rpm for 15 min at 4°C. Equal amounts of protein lysates from different treatment samples were separated by homemade SDS-PAGE gels and transferred onto PVDF membrane. The membrane was then blocked with 5% non-fat dry milk for 1 h at room temperature and probed against specific primary antibody for Nrf2, GCLC, GSR, PTEN, Actin, GAPDH, tubulin. After washing the membranes were incubated for 1 h with corresponding peroxidase-conjugated secondary antibody. Washed blots were immunodetected using Supersignal West Pico Chemiluminescent substrate kit (ThermoScientific, Rockford, IL). GAPDH, Actin or tubulin expression was used to normalize loading. The immunoreactive signals were quantified by densitometry using Image J software.

**Figure 6:**
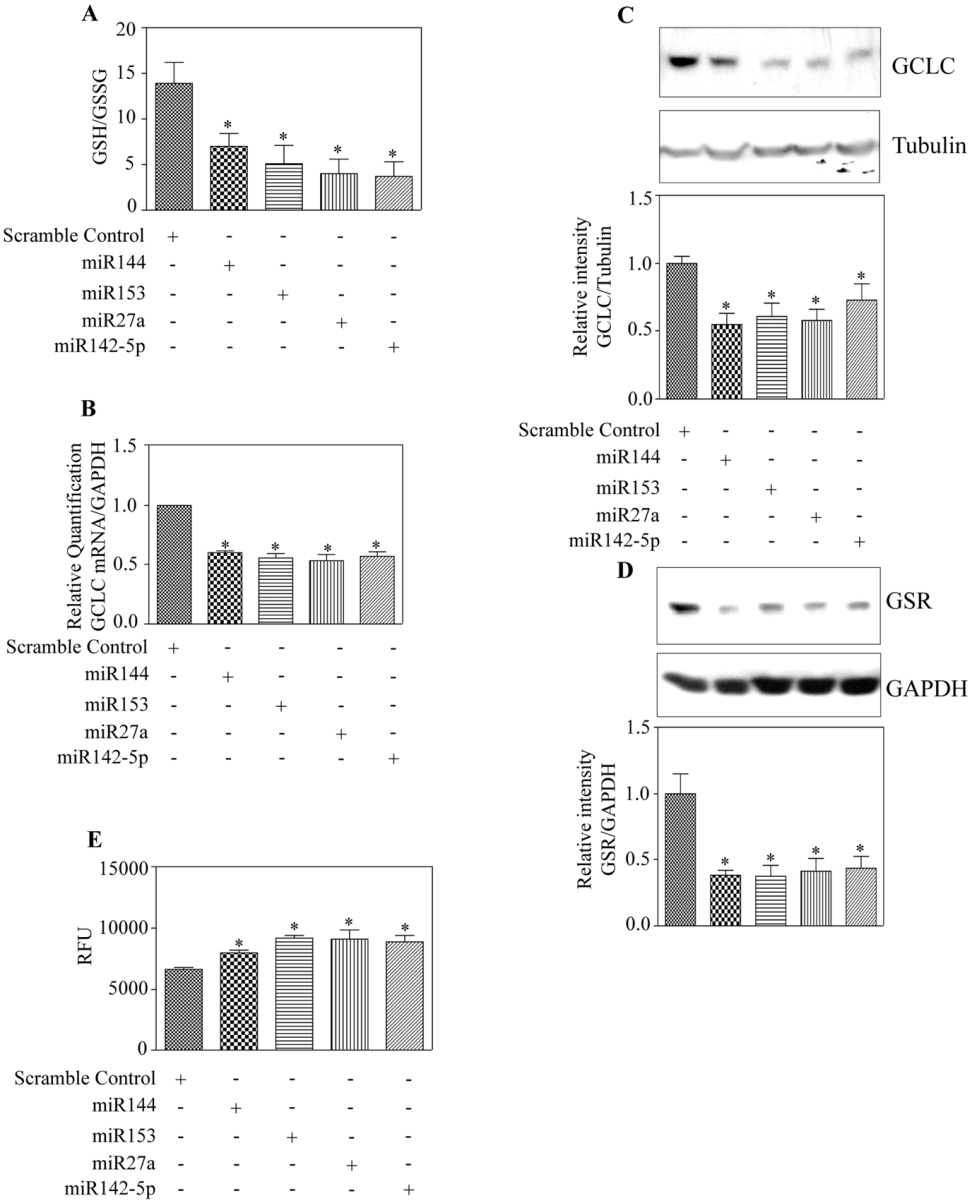
Overexpression of miR144, miR153, miR27a and miR142-5p reduces GCLC and GSR expression affecting GSH/GSSG ratio and cellular ROS levels. (A) SH-SY5Y cells were transfected with indicated miRs and 48 h post-transfection processed for GSH and GSSG measurement as described in “Materials and Methods”. The ratio of relative luminescence for GSH and GSSG from 4 different samples was shown. (B) RNA was isolated and retro-transcribed from cells treated as in 6A, and Taqman based realtime qPCR was carried out using GCLC and GAPDH specific primers. Data is expressed as relative change in GCLC mRNA levels normalized to GAPDH (n = 4). (C) Western blotting was performed on total cellular lysates from the indicated experimental group with GCLC antibody. The corresponding blot was stripped, reprobed with GAPDH antibody and a representative data are shown. Densitometric analysis of GCLC bands normalized to GAPDH bands are shown in the bottom panel (n = 5). (D) Whole cell lysates (similar to 6C) were analyzed for the expression of GSR and GAPDH. A representative Western blot image is shown in the top panel. Graph of densitometric scans of GSR immunoblot normalized to that of GAPDH is presented in the bottom panel (n = 4). (E) Different miR mimics were overexpressed as indicated. Cellular ROS production was measured using ROS-sensitive probe, DCF-DA and fluorescence signal of DCF formed was recorded by GLOMAX Multidetection system. Relative fluorescence units were plotted (n = 4). In panel (A-E), * p<0.05 as compared to scramble miR transfected group by one way ANOVA and Newman Keuls post-test.

### Cytosolic and Nuclear Extraction

Cytosolic and nuclear fractions of SH-SY5Y cells were extracted using a commercially available NE-PER Nuclear and Cytoplasmic Extraction kit (ThermoScientific, Rockford, IL) according to the manufacturer’s protocol. Effects of various microRNA treatments on Nrf2 levels in the cytoplasmic and nuclear fractions were determined using immunoblotting. Purity of cytosolic and nuclear fraction was determined by the expression of GAPDH and lamin b1 respectively.

**Figure 7:**
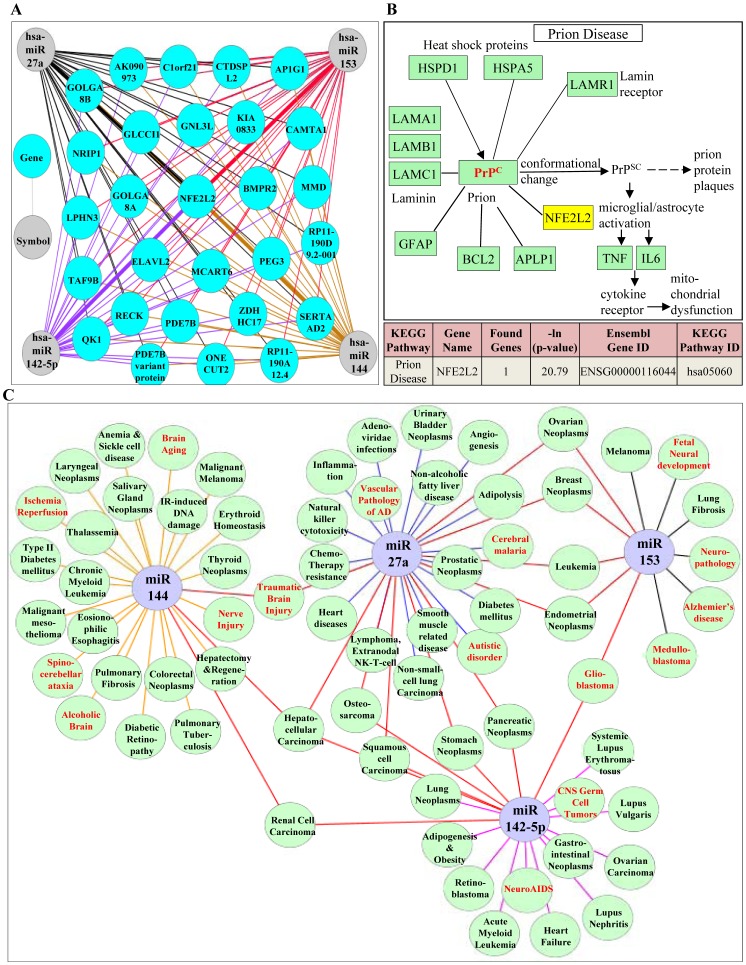
*In-silico* based identification of Nrf2 dependent molecular pathway and complex network of disease processes that could be regulated at the intersection of miR144, miR153, miR27a and miR142-5p. (A) “mirDIP” based computational analysis of tested miRs (miR144/153/27a/142-5p) and identification of number of genes that could be potentially targeted at the intersection of the 4 miRs. mirDIP generated list was used as an input file and a network was generated using another bioinformatic program, “Cytoscape_v2.8.2″. NFE2L2, which is at the intersection of 4 miRs is represented by a bold colored lines. While all the other target genes which are at the intersection of 4 miRs are represented by independent colored lines (miR144-brown; miR153-red; miR27a-black; miR142-5p-purple). (B) KEGG pathway mapping identified Prion disease as the predominant pathway at the intersection of miR144, miR153, miR27a, miR142-5p involving NFE2L2, one of the genes that is predicted to be regulated with a high –ln(p-value) of 20.79 by “DIANA-mirPath”. (C) Using the concept of network biology, we constructed miRNA-associated disease network (MDN) to visualize the relationship of miRs of our interest with multiple pathologic/pathogenic conditions. The network was generated by compiling the data manually from Pubmed (till 05/07/2012) along with the information from two other databases, Human miRNA Disease Database (HMDD - http://202.38.126.151/hmdd/mirna/md/) and miR2Disease (http://www.mir2disease.org/). This compiled data was used in “Cytoscape_v2.8.2″ to establish connections between miRs and associated diseases. Different diseases associated with indicated miRs are represented as light green colored nodes. Diseases associated with individual miRs are connected by specific colored edges (lines) and any two different miRs that share one common disease are connected by red colored edge. Neuro specific pathologies are represented in red font.

### RNA Extraction and Taqman Based Real-time q-RT PCR

Total cellular RNA was extracted using Trizol reagent (Invitrogen, Carlsbad, CA). 1.5 µg of RNA was incubated with gDNA wipe out buffer (Qiagen, Valencia, CA) at 42°C for 2 min to remove any genomic DNA contamination. Following gDNA elimination, the RNA is reverse transcribed to cDNA using Quantitect reverse transcription kit according to instructions (Qiagen).

For real time PCR analysis of Nrf2, NQO1, GCLC, endogenous Nrf2 3′ UTR and GAPDH mRNA expression, 1/10^th^ of cDNAs prepared as above was used. Taqman gene expression assays consisting of predesigned primer and probe sets specific for human NFE2L2 (Hs00232352_ml); human NQO1 (Hs02512143_s1); human GCLC (Hs00155249_m1); human GAPDH (Hs03929097_g1) and custom designed for human endogenous Nrf2 3′ UTR (AJGJPXK) were from Applied Biosystems (Bedford, MA). Real time PCR amplification was performed in 386-well optical plates in a final volume of 20 µL containing 10 µL of TaqMan Universal Mastermix (Applied biosystems), 20 pmol of respective primers and 1/10^th^ of reverse transcribed RNA. Real time PCR was conducted on a Biorad CFX384 Real time system (Biorad). The thermal cycling conditions used consisted of 50°C/2 min; 95°C/10 min followed by 40 PCR cycles at 95°C/15 sec and 60°C/1 min. Fold change in the mRNA expression was calculated on the basis of cycle threshold (Ct) value and GAPDH was used for normalization. Relative quantitation of transcript levels were plotted as fold difference as compared with untreated samples and was calculated by the formula: 2^−ΔΔCT^, where ΔCT value of the sample was arrived at by subtracting the Ct value of respective genes from the Ct value of GAPDH and ΔΔCT value was determined by subtracting ΔCT value of treated condition from ΔCT of untreated condition.

miRNA detection by real time analysis involved reverse transcription of cDNA using a small RNA specific stem-loop RT primer (hsa-miR144: RT-002676; hsa-miR153: RT-000476; hsa-miR27a: RT-002445; hsa-miR142-5p: RT-002248; U6 snRNA: RT-001973). Once specific cDNA was generated, individual miRNA was detected using Taqman small RNA assay Real time PCR analysis (hsa-miR144: TM-002676; hsa-miR153: TM-000476; hsa-miR27a: TM-002445; hsa-miR142-5p: TM-002248; U6 snRNA: TM-001973). Results were normalized to small nuclear RNA U6 that served as control and the data was expressed as Log 2 fold change in respective miRs/U6 snRNA levels.

### Luciferase Assays

SH-SY5Y cells were transfected with various constructs as indicated in appropriate figures. 48 h post transfection, cellular extracts were collected after lysing with passive lysis buffer (Promega, Madison, WI) and centrifuging at 15,000 rpm for 20 min. Firefly and Renilla luciferase activities were determined from lysates using dual luciferase assay system (Promega, Madison, WI) in a Glomax (20/20) luminometer. As suggested by the manufacturer, firefly luciferase activity was normalized either to Renilla luciferase activity or protein concentration obtained from the corresponding samples.

### Site-directed Mutagenesis using Overlapping Primers

Luciferase reporter constructs containing mutation of miR153, miR27a and miR142-5p target sites of Nrf2 3′ UTR were generated using partial overlapping primer based PCR according to Zheng et al. [25]. Primer pairs used in this strategy were designed to possess complementarities to each other at 5′ terminus (bases underlined in Table 1). Briefly, PCR amplifications were performed using PrimeSTAR Max DNA polymerase, a unique high-performance DNA polymerase with wild type Nrf2 3′ UTR reporter construct (pGL3 WT Nrf2 3′ UTR) as template. The PCR parameters were initiated by pre-heating the reaction components to 94°C for 3 min. Cycling parameters for amplifying mutant miR153 include 30 cycles of 98°C/15 sec, 60°C/15 sec, and 68°C/1 min; followed by extension at 68°C/7 min. Individual mutants of 27a and 142-5p were PCR amplified using 25 cycles of 98°C/15 sec, 65°C/15 sec, and 72°C/45 sec; followed by extension at 72°C for 7 min. PCR products carrying the appropriate mutations were gel electrophoresed, purified, DpnI digested at 37°C for 1.5 h and inactivated at 80°C for 20 min. An aliquot was transformed into NEB10-beta competent E.coli cells and a total of 7 colonies from each were selected and their plasmids were isolated by PureYield Plasmid miniprep kit (Promega, Madison, WI). All mutants were sequence verified for the presence of desired mutations and absence of any other mutations in the Nrf2 3′ UTR sequence (Genewiz, South Plainfield, NJ).

### GSH/GSSG Glo Assay

Luminescence based GSH/GSSG Glo assay was used to measure the GSH/GSSG levels. SH-SY5Y cells transfected with indicated miRs for 48 h and processed for total glutathione (GSH) and oxidized glutathione (GSSG) in parallel reactions according to manufacturer’s instructions (Promega, Madison, WI) with minor modifications. Following treatment, the cells were scraped in GSH buffer and halved into two fractions equally. Immediately one half of the lysate was added to a tube containing water and the other half of the lysate was added to N-Ethylmaleimide (NEM) containing tube that blocks glutathione in its reduced state. Intact GSSG that is left unblocked by NEM was then reduced to GSH followed by addition of luciferin generation reagent containing DTT and glutathione-S-transferase. The resultant luciferin formed in a GST-coupled firefly luciferase reaction was measured in terms of luminescent signal in a Glomax Multi detection system (Promega, Madison, WI). Both GSH and GSSG levels were determined against standard GSH according to manufacturer’s instructions. GSH and GSSG levels were normalized to protein concentrations and the data were plotted as GSH/GSSG ratio.

### ROS Detection by Fluorimetry and Imaging

SH-SY5Y was transiently transfected with indicated miRs as explained earlier. 48 h post-transfection, old media was replaced with PBS containing 25 µM 2′,7′-dichlorofluorescein (DCF-DA) and incubated at 37°C for 30 min in dark conditions. Cells were then gently washed with PBS and scraped with PBS containing 2 mM EDTA. The samples were transferred to a 96-well clear bottom-white walled plate and fluorescence intensity was measured on GLOMAX Multi Detection System with Optical Kit Blue (Excitation 490 nm and Emission 510–570).

For imaging, cells were treated with 5 µM of DCF-DA at the end of the experiment in dark and returned to incubator. After 30 min the cells were gently washed with PBS to remove excess fluorophore. Cells were imaged immediately on an Olympus IX71 microscope with a 40× objective. DCF was excited at 488 nm using a multi-Argon laser and the emission collected through 510 nm barrier filter. The laser intensity output used was attenuated to minimize photobleaching and phototoxicity to the cells during XY scanning by adjusting PMT, gain and offset. For reproducibility and comparison purposes, all experimental conditions as well as microscope settings were kept identical. Random fields were imaged and a representative photomicrograph was presented.

### Statistical Analysis

Results are expressed as means±s.e.m. For experiments involving more than two groups, statistical evaluation of the data was performed using one-way ANOVA followed by Newman-Keuls as post-test analysis [2]. Student’s t-test was performed in case of experiments with two groups. A value of P<0.05 was considered as statistically significant.

## Results and Discussion

### *In-silico* Prediction of Target miRNAs against Human Nrf2 and Experimental Validation of Nrf2 Downregulation by Forced Expression of miR144, miR153, miR27a and miR142-5p

Nrf2, an essential transcription factor for regulating both basal and inducible expression of diverse cytoprotective genes [26], [27] has been recently demonstrated to be regulated by miR144 and miR28 in non-neuronal models [22], [23]. It is well known that multiple miRs target a single gene and a single miR can regulate many genes [28], [29] and hence to obtain a deeper understanding of complex gene networks a comprehensive knowledge of a target-miRNA interaction is in place. Taking these findings into account we sought to examine the miR based Nrf2 regulation in neuronal cellular model. To test this hypothesis, we primarily employed bio-informatic analysis of human Nrf2/NFE2L2 3′ UTR for miRNA seed sequences using the “predicted targets component of miRecords”. Nrf2 transcript harbors a 3′ UTR of ∼428 bp in length and hence many potential miRNA binding sites are possible. As expected the search resulted in overall prediction of 408 plausible miRNA that could target human Nrf2. Typically the accuracy of any individual prediction program has been calculated to range between 30–76% [30]–[32]. This necessitates selection of the right database before experimental testing and to this end the first level of filter we applied was choosing “miRecords” which integrates data from 11 different established miRNA target prediction programs. The second level of stringency applied was selecting only the top and common miRs which remained prominent at the intersection (overlapping part) of at least 6 individual databases listed in miRecords. Based on these criteria, we narrowed down to a list of 4 different miRs (hsa-miR27a, hsa-miR153, hsa-miR142-5p including the already reported hsa-miR144) (Fig. 1A). Having selected the putative miRs that could target Nrf2, we next assessed for the conservancy of those seed sequences of miRs in Nrf2 3′ UTR among different organisms by “TargetScan”. It is clear that the different miRNA target sequences on Nrf2 3′ UTR display a high phylogenic conservation by ∼90% among various mammals (Fig. 1B). This likely indicates a critical role for these miRNA binding sites on Nrf2 3′ UTR to regulate Nrf2 that is conserved during a highly dynamic evolutionary process. To validate whether the computationally predicted miRNAs could target Nrf2 in neuronal system, we chose human neuroblastoma SH-SY5Y, a neuronal-like subline of SK-N-SH cells and overexpressed with each of these miRs (miR144, miR153, miR27a and miR142-5p) individually. These cells possess the phenotype of sympathetic ganglion neurons and have been extensively utilized to study gene expression changes against neurotoxicants and various other neuro-related phenomena such as oxidative cytotoxicity, neuronal apoptosis, neurodegeneration, neurotransmitter release and calcium homeostasis [33]–[39]. Primarily, 48 h post-transfection, the transfection efficiency of these miRs was evaluated by Taqman based qRT-PCR (Fig. S1A–S1D). Endogenous level of miR144 and miR142-5p in SH-SY-5Y cells were observed to be negligible. Thus, in order to calculate the transfection efficiency of these miRs, a widely accepted method of imputing a Ct threshold value of 40 was adopted [40]–[42]. Results were analyzed and expressed as natural logarithm (ln) of relative quantity of miR144 and miR142-5p, normalized to U6 snRNA from a calibrator sample (scramble control miR) [42], [43]. Transfection of miR144 and miR142-5p duplex increased the endogenous level of these miRs by log (2) 3.4 fold and 5.2 fold respectively (Fig. S1A; S1D). The level of miR153 and miR27a was relatively high when compared with endogenous levels of miR144 and miR142-5p. Ectopic expression of miR153 and miR27a resulted in log (2) 8.5 fold and 8.8 fold increase in levels of these miRs respectively (Fig. S1B; S1C). Having confirmed the efficiency of miR overexpression, we next performed Western blot analysis for Nrf2 using anti-Nrf2 (C-20; sc-722). It is to be noted that the actual predicted molecular mass of Nrf2 protein is 68 kDA and previous studies indicate that due to its inherent high acidic charges, Nrf2 anomalously migrates at ∼100 kDA in SDS-PAGE [6]. Other reports also suggest that ∼100 kDA form of Nrf2 is a result of dimer formation with actin [44] or ubiquitination of Nrf2 [45]. A recent report from Lau et al., [46] concluded ∼95–110 kDA as the appropriate molecular weight of Nrf2. However, there are many independent reports including a study wherein bacterially expressed recombinant TAT-Nrf2 protein was purified and identified to migrate as 72 kDA protein in SDS-PAGE (∼69 kDA −Nrf2+∼3 kDA TAT Tag) [47]–[51]. In our experiments, overexpression of all of the aforementioned miRs individually decreased both the expression of predicted (68 kDA) and apparent Nrf2 protein (that runs at ∼100 kDA) significantly by ∼50% (P<0.05) when compared to scramble miR transfected SH-SY5Y cells (Fig. 1C; 1D). Both 100 kDA and 68 kDA Nrf2 bands were normalized to GAPDH level following Image J quantification. We confirmed the specificity of these miRs by overexpressing SH-SY5Y cells with a non-Nrf2 targeting microRNA, miR21 (predicted by miRecords). As expected, miR21 overexpression did not result in any change in Nrf2 protein expression (Fig. S2A). miR21 transfection was validated by significant decrease in protein levels of one of its bonafide targets, PTEN (Fig. S2B). Overall, this data suggests that Nrf2 is translationally repressed by miR144, miR153, miR27a, miR142-5p in a specific manner. Also, at this point, the possibility of these miRs altering the abundance of available Nrf2 mRNA pool for translation is not ruled out as miRs have been shown to regulate gene expression by mRNA deadenylation and decay [52], [53].

### miR144, miR153, miR27a and miR142-5p Represses Nrf2 3′ UTR and Endogenous Nrf2 mRNA

MicroRNAs repress protein production by preferentially interacting with complementary sequence motifs in the 3' untranslated region (UTR) of target mRNAs. It has been previously demonstrated that human Nrf2 3′ UTR possess 2 potential miR144 binding sites at 265–271 and 370–377 [22]. According to our prediction analysis using TargetScan, we observed evolutionarily conserved binding sites for miR27a, miR142-5p, miR153 between 62–68, 83–90, 98–105 respectively in the human Nrf2 3′ UTR (Fig. 1B). Thus, to test whether the forced expression of selected individual miRNA candidates (miR144, miR153, miR27a and miR142-5p) have any repressing effect on Nrf2 3′ UTR, we used a reporter construct that was cloned with 428 bp of human Nrf2 3′ UTR downstream of luciferase gene. Typically, a decreased luminescence output in this assay indicates that miRNAs had effectively bound to and targeted the 3′ UTR. It was observed that forced expression of all the tested miRs significantly repressed Nrf2 3′ UTR reporter activity (p<0.05). The results show that individual overexpression of miR144, miR153, miR142-5p effected a ∼42% repression and a maximal repression by about ∼68% was shown by miR27a (Fig. 2A). Though ectopically expressed 3′ UTR transcriptional reporter construct is used to estimate the expression pattern of a gene that is regulated by miRs, it may not reflect the precise regulation that occur in endogenous cellular mileu. The cloned UTR may lack several flanking cis-regulatory elements (or) the intrinsic Nrf2 coding sequence that dictates RNA fold necessary for miRNA interaction as opposed to the endogenous set. To eliminate such experimental bias, Bartuma et al. [54] employed RT-PCR based detection of endogenous 3′ UTR of HMGA2 in various fetal tissues, adipocytic tumors and amniocytic cell cultures. We adopted this strategy to assess the effect of overexpression of indicated miRNA candidates on endogenous expression of Nrf2 3′ UTR mRNA using Nrf2 3′ UTR specific primers by real time qRT-PCR analysis. As shown in Figure 2B, overexpression of different precursor miRs showed significant reduction in endogenous expression of Nrf2 3′ UTR mRNA when compared to that of scramble miR (p<0.05). To further confirm that Nrf2 3′ UTR regulation by miR144, miR153, miR27a, miR142-5p indeed impact the expression of Nrf2 mRNA, we determined the levels of Nrf2 message in SH-SY5Y cells transfected with and without the aforementioned miRs using quantitative real time PCR for Nrf2. The cells transfected with precursor miR144, miR153, miR27a, miR142-5p displayed significant reduction in Nrf2 transcript levels when compared with scramble miR transfected cells (p<0.05) (Fig. 2C). Though, the endogenous level of Nrf2 3′ UTR and Nrf2 mRNA was significantly repressed by ectopically expressing miRs, the magnitude of reduction was not of similar extent (Fig. 2C vs 2B). The probable reason is that at a given time, the cellular mRNA pool will include both species of Nrf2 mRNA namely, complete Nrf2 (with coding sequence+UTR intact) and Nrf2 (with coding sequence+partial UTR: in the process of decay). Thus, the primers used in Nrf2 mRNA detection by real time analysis would measure all the species of Nrf2 mRNA accounting for actual fold in reduction. However, the primers used in Nrf2 3′ UTR analysis would detect only the species with intact UTR sequence, in otherwords it would not detect the pool of mRNA where UTR sequence is lost. In addition, the significant reduction in Nrf2 mRNA enforced by miRs is not precisely reflected in the magnitude of Nrf2 protein reduction (compare ∼50% in protein repression; Fig. 1D vs ∼25% in mRNA levels; Fig. 2C). Though, several studies show miRNA induced protein repression strongly correlates to mRNA levels [53], [55], [56], the possibility of miRs inducing translational repression even before mRNA deadenylation and decay have been very recently reported [52], [57]. Thus, future studies should assess the relative timing and involvement of various closely linked events such as translation repression, mRNA deadenylation and decay in miR144/miR153/miR27a/miR142-5p induced silencing of Nrf2. Altogether, our results strongly suggest that miR144/153/27a/142-5p could suppress Nrf2 gene expression through 3′ UTR binding and down-modulating Nrf2 mRNA in SH-SY5Y neuronal cells.

### Enforced Expression of miR(s) Affects Nrf2 Localization and its Transactivation

To test whether our findings of decreased Nrf2 protein in whole cell homogenates would affect its localization and function we carried out cytosolic and nuclear fractionation and analyzed for Nrf2 expression by immunoblotting. Nrf2 corresponding to ∼100 kDA and 68 kDA was found to be decreased in both the cytosolic and nuclear fractions of tested miRNA mimics transfected cells when compared to scramble control pre-miR transfected cells (Fig. 3A & 3B). Blots were stripped and reprobed for GAPDH and laminb1 that served as loading controls. Further the fraction purity was ascertained by reverse probing the cytosolic blot for laminb1 and nuclear blot for GAPDH. Generally, a low level of nuclear Nrf2 results in declined basal or induced transactivation of its target genes [26], [27]. We therefore tested whether the miRNA induced downregulation of Nrf2 protein results in decreased mRNA expression of one of the “Nrf2 regulons”, NQO1. Taqman based real time qRT-PCR analysis with NQO1 gene specific primers demonstrated that overexpression of miRs resulted in significant reduction in expression of NQO1 mRNA (p<0.05) (Fig. 3C). To confirm if the expression of NQO1 mRNA decreased by overexpression of miRs was due to dysregulated transcriptional activation, we performed a luciferase based reporter assay by co-transfection of plasmid containing NQO-1 ARE enhancer element along with the indicated miRs. Overexpression of miRs resulted in a profound decrease by about 7 fold in NQO1 ARE – driven luciferase activity (Fig. 3D) indicating the effective suppression of Nrf2-mediated transactivity. Though we observed a remarkable downregulation of NQO1-ARE activity (Fig. 3D), a comparable level of reduction in nuclear Nrf2 levels was not noted (Fig. 3B). The evident disparity could be argued for the fact that luciferase assay depends on the degree of binding of active transcription factors (TFs) (herein, active Nrf2, but not total Nrf2 level) to its consensus element. While, Western output is dependent on the level of TFs rather than the state of TFs (active or inactive) and its degree of binding to its target. Further, strict correlation among ARE reporter activity and ARE containing endogenous target expression (mRNA levels of GCLC, NQO1) was also not observed. It is understood that 4× NQO1-ARE reporter plasmid is engineered with only Nrf2 binding elements in which case the activity of reporter plasmid is solely dependent on Nrf2. However, in the endogenous cellular setting, target gene expression could be dependent on the enhancers and suppressors other than Nrf2 which could bind to non-ARE DNA regions. Therefore, the luciferase based transcriptional activity measurement of NQO1-ARE construct would be very sensitive to Nrf2 levels as opposed to endogenous transcriptional activity measurement (mRNA levels by real time) and thus, connecting these two different measurement strategies based on exact magnitude of changes would be practically difficult. Nevertheless, our findings demonstrate that in neuronal SH-SY5Y cells, miR144/153/27a/142-5p induced Nrf2 downregulation affects the nucleo-cytoplasmic concentration of Nrf2 which is reflected in its inefficient transactivating ability.

### miR144/miR153/miR27a/miR142-5p Mediated Repression of Nrf2 is Keap1-independent

Keap1 mediated control of Nrf2 is a spatiotemporally regulated process [58] and cytosolic Keap1 is believed to inversely control the nucleo-cytoplasmic shuttling of Nrf2 and latter’s access to its targets in nucleus [4], [59], [60]. Notably it has been demonstrated that miRs can also induce translation upregulation of target mRNAs on certain instances [61], [62]. Thus, we raised a critical question as to whether overexpression of the indicated miRs has any possible auxiliary impact in upregulating Keap1 thereby indirectly repressing Nrf2. To assess this, we first performed a bio-informatic analysis using “miRecords” for Keap1. Unlike Nrf2 analysis which was performed with a high stringency of mapping the top miRs from atleast 6 individual databases (Fig. 1A), miR:Keap1 prediction was carried out with less stringency and selected those miRs that were populated at least in 2 different databases. 124 miRNA was predicted to target Keap1 (NM_012289) and a representation of miR-Keap1 network generated by “Cytoscape_v2.8.2” is shown (Fig. S3). It is clear from the list that even with less stringent filtering, Keap1 is not predicted to be a regulatory target of any of the 4 miRs tested herein. This suggests that Nrf2 regulation by miR144/153/27a/142-5p appears to be a tightly controlled event with the likelihood of circumventing any redundancies involving Keap1. Having scanned the *in-silico* analysis of Keap-1:miR network, we next validated the bioinformatic prediction using immunoblotting for Keap1 in total cellular lysates. Overexpression of the miR mimics did not affect Keap1 protein levels relative to that of scramble control miR mimic (Fig. 4). Recently, Keap1 independent regulation of Nrf2 by miR28 has been reported in breast cancer cells [23]. However, till date there is no report on miR based Nrf2 regulation in neuronal system. Thus our study is the first to demonstrate that Nrf2 protein could be subjected to translation repression by miR144/miR153/miR27a/miR142-5p in a Keap1 independent manner in neuronal cellular system.

### Nrf2 is a Direct Target of miR144, miR153, miR27a and miR142-5p

As we showed that the miRs can downregulate Nrf2 3′ UTR expression thereby Nrf2 protein production in a Keap1 independent manner (Fig. 2 & Fig. 4), we next sought to determine whether this is indeed due to a direct effect of these miRs via binding to its respective complementary sequence on the 3′ UTR. To address this specificity of these interactions, we generated mutation reporter constructs bearing a 3-nucleotide change in the individual miR target sequences on Nrf2 3′ UTR by site directed mutagenesis and compared its activity against the WT Nrf2 3′ UTR. The schematic representation of binding sites and the individual mutants for miR144 (site-1 & site-2), miR153, miR27a and miR142-5p in the human Nrf2 3′ UTR was shown in Fig. 5A and the successful incorporation of mutagenized bases was confirmed by sequencing of the individual mutant constructs. We focused on miR144 (site 2) in the current study as Sangokoya et al. (2010) [22] previously demonstrated that miR144 binding site 2 (position 370–377) is only involved in miR144 mediated repression of Nrf2 in erythrocytes. All the constructs either wild type or indicated mutants of Nrf2 3′ UTR were co-transfected into cultured SH-SY5Y cells along with individual miR mimics and a renilla luciferase transfection control vector. After 48 h of transfection, both firefly and renilla luciferase levels were assayed by luminometry. Transfection of WT Nrf2 3′ UTR construct along with individual miRs significantly repressed the luciferase activity (p<0.05) (Fig. 5B–5E; compare lane 1 vs lane 2). In each case, mutation of miR144 (or) miR153 (or) miR27a (or) miR142-5p binding sites on Nrf2 3′ UTR failed to downregulate the luciferase activity as opposed to those observed in WT type reporter construct (Fig. 5B –5E; compare lane 3 vs lane 4). These experiments demonstrate that in order for these miRs to repress Nrf2 activity, the respective miR binding sites on Nrf2 3′ UTR must be intact. In other words, mutation of individual miR binding sites viz. miR144, miR153, miR27a and miR142-5p abrogates the interaction and binding of corresponding miRs to human Nrf2 3′ UTR, thus indicating Nrf2 as a direct regulatory target of these miRs.

### Ectopic Expression of miR144/153/27a/142-5p Deregulates GSH and ROS Levels

The critical role for Nrf2 in regulating ARE/GSH pathway suggests that any impairment in Nrf2 levels induced by miR144/153/27a/142-5p may impose damaging effects on GSH homeostasis.

Therefore, we explored whether overexpression of these miRs could have any role in modifying GSH/GSSG ratio, a monitor of cellular antioxidant status. Importantly, a decrease in GSH/GSSG ratio can also be used as an indicator to distinguish the oxidatively stressed cells from non-stressed cells [63]. Luminescence based GSH/GSSG glo assay revealed that exogenous expression of miRs resulted in ∼2 fold decrease in the ratio of GSH/GSSG (p<0.05) (Fig. 6A). We next assessed whether changes in GCLC, a rate limiting enzyme involved in *de novo* synthesis of GSH is the contributing factor for reduced GSH/GSSG ratio. GCLC transcript expression by real time qRT-PCR analysis indicated a 1.5 to 2 fold decrease in different tested miRs as compared to scramble control miR (p<0.05) (Fig. 6B). Consistent with changes observed in GCLC message, protein levels of GCLC was also significantly altered (p<0.05) (Fig. 6C). In general, glutathione reductase (GSR) is an enzyme that maintains glutathione pool in the reduced form in cytosol. Thus any impairment in GSR coupled with oxidative stress would be expected to favor accumulation of oxidized glutathione (GSSG). Hence, in our experimental conditions we assessed whether expression of GSR is altered and indeed we observed that overexpression of all the tested miRs significantly repressed GSR protein by 1.5–2 fold from that of scramble miRs (p<0.05) (Fig. 6D). It was previously reported that Nrf2 dysregulation significantly altered the expression of GCLC and GSR affecting GSH homeostasis in Nrf2 knockout mice and mammalian cells [2], [64], [65]. In our present study, alterations in Nrf2-dependent *de novo* pathway (involving GCLC) and regeneration pathway (involving GSR) both are likely to contribute to diminution of GSH/GSSG ratio. Nrf2 deficiency has been reported to increase accumulation of oxidized form of glutathione [64] as well as intracellular ROS levels [2], [66]. Given that all the 4 tested miRs reduced Nrf2 levels and affected GSH homeostasis, we next tested whether this could be related to increased ROS levels by DCF-DA based imaging and fluorescence assay. DCF-DA is a non-polar, cell-permeable, sensitive flurophore that is widely used to detect several ROS [67], [68]. DFC-DA is converted to DCF, a fluorescent product, only in presence of ROS and the intensity of DCF fluorescence reflect the levels of ROS products in cellular system. Overexpression of various test miR mimics resulted in a moderate, yet significant (p<0.05) increase in intracellular DCF fluorescence ranging from ∼20% to 37% compared with scramble control miR (Fig. 6E). Further, image analysis of ROS also yielded an identical result as that of semi-quantitative fluorescence assay (Fig. S4). Thus, in view of preserving redox potential that is key to a normal cellular physiology, our results suggest that Nrf2 dependent redox homeostasis could be controlled in this neuronal system by regulation of levels of the following miRs: miR144/miR153/miR27a/miR142-5p.

### Nrf2 at the Intersection of 4 Different miRs. Construction of miR-disease Network (MDN) with Respect to miR144/miR153/miR27a/miR142-5p

As one to many miR:target relationships are likely, we next analyzed the possible strong candidates including Nrf2 that could be mapped at the intersecting points of miR144, miR153, miR27a and miR142-5p using “mirDIP (microRNA: Data Integration Portal)”. Besides the default settings in mirDIP, we applied a secondary level of filter by assigning a standardized score of minimum “50.0” and searched for targets of chosen 4 miRs with microRNA.org as database criteria and intersecting IDs. Totally 28 genes were computed to be at the intersection of 4 different miRs (hsa-miR144/hsa-miR153/hsa-miR27a/hsa-miR142-5p). This list was used as an input file and a network of targets:(144∩153∩27a∩142-5p) was generated by bioinformatic interaction network generating software “Cytoscape_v2.8.2” (Fig. 7A). In order to visualize the collective effect of co-expressed miRs in modulating specific pathways, we performed simultaneous enrichment analysis using “DIANA-mirPath” that considers multiple miR:target relationships alongside comparing each set of miRNA targets to all known KEGG pathways. This software has an integrated chi-square testing component and following the analysis results are displayed as a negative natural logarithm of the enrichment p-value which is distinct for each pathway. Higher the p value, stronger the association of a particular gene with an indicated KEGG pathway. It is to be noted that DIANA-mirPath analysis populated NFE2L2 as the principal gene at the intersection of miR144, miR153, miR27a, miR142-5p with a highest –ln(p-value) of 20.79 that is mapped to involve in Prion disease by KEGG pathway (Fig. 7B). The computationally generated intersection dataset which gives an overview of the cooperative downregulation of Nrf2 by these miRs was confirmed by overexpressing combination of these 4miRs at 1/10^th^ of the concentration that were tested individually. Interestingly, we observed a striking and similar level of downregulation of Nrf2 on combining these miRs even at a low dose (40 nM–10 nM each) (Fig. S5A) as opposed to 100 nM used in individual miR transfection experiments. Further, 40 nM of combined miRs (10 nM each) resulted in significant downregulation of GSH/GSSG ratio which is almost comparable to that of cells overexpressed with 100 nM concentration of individual miRs (Fig. S5B). In general, these miRs (miR144, miR153, miR27a, miR142-5p) when individually present can regulate numerous targets/pathways and the effect of particularly targeting Nrf2 may vary from moderate to high depending on the cellular/stressor setting. In order to facilitate the comparison of different biological processes that are altered in the aforementioned miRNA datasets, enrichment p-value analysis of the Union dataset with all these miRs was also performed and the results are presented in a bar plot graph (Fig. S6). It is clear that in most of the top targeted pathways, the Union dataset –lnP (Red bars) are higher than the –lnP values obtained for each single miRNA (Yellow, Green and Blue bars) (Fig. S6) indicating that co-ordinated dysregulation of most of the Nrf2 dependent biological pathways might be directly proportional to co-expression of these miRs. The results of Fig. S5 and S6 suggests that in a given cellular context, when these candidate miRs: miR144, miR153, miR27a and miR142-5p co-exist even at low levels, each would bind to Nrf2 via multiple, distinct binding sites and may perhaps increase the robustness and likelihood of targeting Nrf2 and its associated functions. In general, identification of disease-related miRs is vital for understanding the pathogenesis of diseases at the molecular level, and to this end, computational analysis of miRNA-disease associations prioritizes and narrows down the candidate miRs with respect to a selective target for further experimental evaluation. Thus, we have presented a comprehensive miRNA:disease linkage network with respect to miRs144/153/27a/142-5p (Fig. 7C). This extended approach is generated based on the published reports gained from experimental evaluations rather than just prediction analysis which clearly suggests that the candidate miRs are strongly associated with heterogenous disease phenotypes with some of the miRs sharing a common disease among them (Fig. 7C). Notably, these miRs either individually or as a combination have been implicated in many neuroabnormalities (Red and Bold letters; Fig. 7C). Given a strong association for oxidative stress in these neurological disorders, our results could suggest a plausible role for these miRs-Nrf2 pathway interactions.

### Conclusions

In conclusion, the results described in this report suggest a role for novel miR candidates viz. miR153, miR27a, miR142-5p and miR144 in regulating Nrf2 expression in SH-SY5Y neuronal cells. Further, miRs tested in the current study impair Nrf2 dependent redox homeostasis in neuronal cellular model which suggests a possible role for these miRs in “redox sensing” and neuropathologies that are ROS dependent. Notably in several stress conditions Nrf2 is activated as a counter-attack response [2], [69], [70]. However this Nrf2 dependent adaptive response is not persistent whilst it becomes dampened even when the cellular system defies a particular stress or inducer. Though the apparent mechanism for such shifting responses during prolonged stress is obscure at this point, our current study suggests a potential mechanism that can temporally restrain Nrf2 by context specific upregulation of either one and/or combination of miRNAs. Future studies are warranted to identify whether this miRNA signature (miR144/153/27a/142-5p) is characteristic of severe oxidative stress related neuro-pathologies which will open new perspectives for antagomiR based therapy to counteract ROS damaging effects.

## Supporting Information

Figure S1 **Transfection efficiency of individual miRs assessed by Taqman based real time q-PCR analysis.** SH-SY5Y cells were transfected with 100 nM of indicated miRs for 48 h. At the end of the experiment, miRNA was isolated miRVana kit (Ambion Inc., Austin, TX). The expression of each miRs was asssessed by Taqman miRNA predesigned assay from Applied Biosystems. Briefly, cDNA was reverse transcribed using a small RNA specific stem-loop RT primer (for each miRNA tested). Real time PCR analysis was performed using Taqman small RNA assay with specific cDNAs as template. Results were normalized to small nuclear RNA U6 that served as control (Panel A-D). The analyzed data was expressed as Log 2 fold change in respective miRs/U6 snRNA levels. * - indicate significant (p<0.05) when compared with scramble control miRNA as assessed by Student’s t-test (n = 4).
(TIF)

Figure S2 **Effect of overexpression of miR21, an Nrf2 non-targeting miRNA on Nrf2 protein expression.** SH-SY5Y cells were transfected with 100 nM of either a scramble miRNA or a non-Nrf2 targeting miRNA, miR21 for 48 h. Whole cell protein lysates were collected and processed for Western analysis with anti-Nrf2 (Panel A) and anti-PTEN (a bonafide target of miR21) (Panel B). Anti-actin immunoblotting served as loading control. Lower panel to the corresponding Western indicate the densitometric analysis of target protein expression. Student’s t-test was performed to assess the statistical significance. * - indicate significant (p<0.05) and ns indicate – not significant when compared with scramble control transfected cells (n = 4).
(TIF)

Figure S3 **Schema of predicted Keap1 targeting miRs.** Analysis of miRNAs that are predicted to target human Keap1 by “miRecords” and only the intersecting miR IDs from at least 2 individual databases are considered to generate Keap1:miR map using a network generating software “Cytoscape_v2.8.2”.
(TIF)

Figure S4 **Effect of overexpression of different miRs on endogenous ROS levels.** SH-SY5Y cells were transfected with either 100 nM of scramble miRNA or individual miRs for 48 h. At the end of the treatment, cells were treated with 5 µM of DCF-DA in dark and returned to incubator. After 30 min the cells were washed gently with PBS to remove excess fluorophore. Photomicrographs were generated using Olympus IX71 microscope.
(TIF)

Figure S5 **Effect of 10 nM each of combination of miR144, miR153, miR27a and miR142-5p on Nrf2 protein expression.** SH-SY5Y cells were transfected with either 40 nM of scramble miRNA or combination of miRs (10 nM each of miR144, miR153, miR27a, miR142-5p) for 48 h. (A) Nrf2 immunoblotting was performed in whole cell protein lysates with anti-GAPDH serving as loading control. (B) As described in Materials and methods, GSH/GSSG Glo assay was performed using GSH as standard (Promega). GSH and GSSG levels were normalized to protein and results were expressed as GSH/GSSG ratio. * - indicate significant (p<0.05) and when compared with scramble control transfected cells (n = 4).
(TIF)

Figure S6 **Integration analysis of multiple miRNAs (miR144/miR153/miR27a/miR142-5p) to various human pathways by DIANA mirPath.** The effect of co-expression of this miRNA signature is visible in the bar plot graph of the –lnP values. In most of the top targeted pathways, the Union dataset –lnPs (Red bars) are higher than the –lnP values obtained for each single miRNA (Yellow, Green and Blue bars).
(TIF)
