# Supplementary figures and images for: Identification of Novel microRNAs in Post-Transcriptional Control of Nrf2 Expression and Redox Homeostasis in Neuronal, SH-SY5Y Cells
Source: PLoS One. 2012 Dec 7;7(12):e51111. doi: 10.1371/journal.pone.0051111 (PMC3517581; doi:10.1371/journal.pone.0051111)

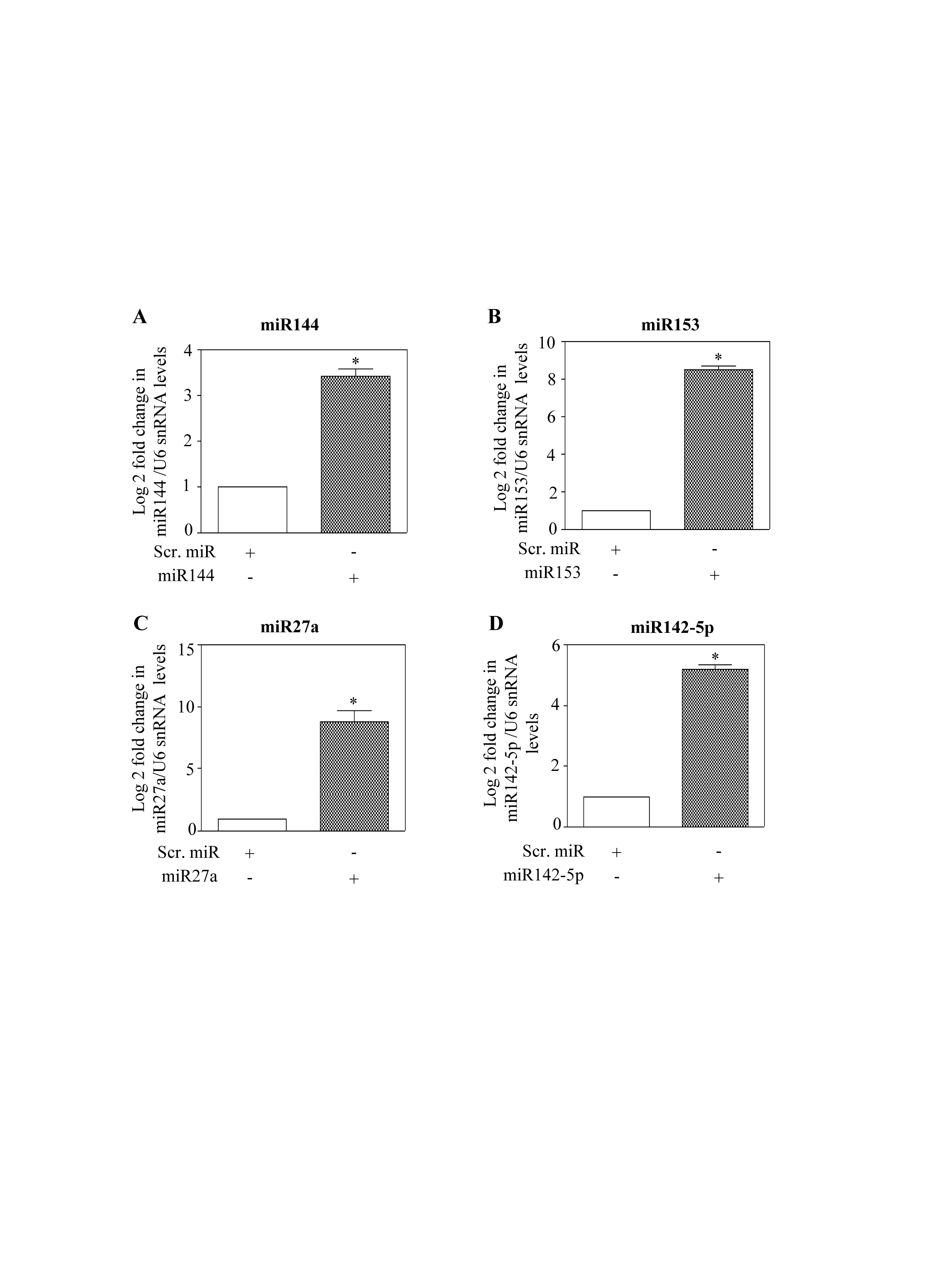

Supplement: Figure S1 — Transfection efficiency of individual miRs assessed by Taqman based real time q-PCR analysis. SH-SY5Y cells were transfected with 100 nM of indicated miRs for 48 h. At the end of the experiment, miRNA was isolated miRVana kit (Ambion Inc., Austin, TX). The expression of each miRs was asssessed by Taqman miRNA predesigned assay from Applied Biosystems. Briefly, cDNA was reverse transcribed using a small RNA specific stem-loop RT primer (for each miRNA tested). Real time PCR analysis was performed using Taqman small RNA assay with specific cDNAs as template. Results were normalized to small nuclear RNA U6 that served as control (Panel A-D). The analyzed data was expressed as Log 2 fold change in respective miRs/U6 snRNA levels. * - indicate significant (p<0.05) when compared with scramble control miRNA as assessed by Student’s t-test (n = 4). (TIF) [file pone.0051111.s001.tif]

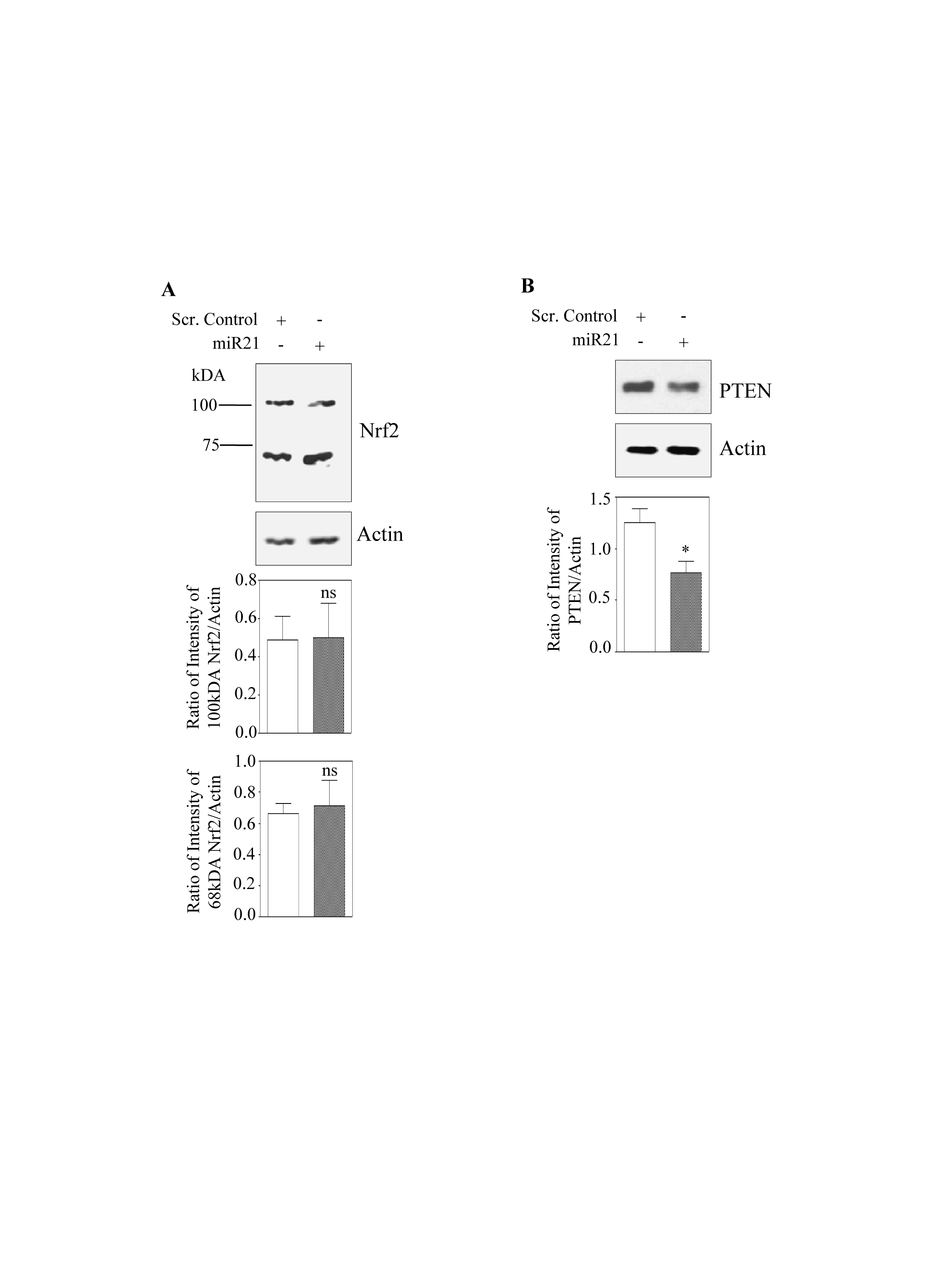

Supplement: Figure S2 — Effect of overexpression of miR21, an Nrf2 non-targeting miRNA on Nrf2 protein expression. SH-SY5Y cells were transfected with 100 nM of either a scramble miRNA or a non-Nrf2 targeting miRNA, miR21 for 48 h. Whole cell protein lysates were collected and processed for Western analysis with anti-Nrf2 (Panel A) and anti-PTEN (a bonafide target of miR21) (Panel B). Anti-actin immunoblotting served as loading control. Lower panel to the corresponding Western indicate the densitometric analysis of target protein expression. Student’s t-test was performed to assess the statistical significance. * - indicate significant (p<0.05) and ns indicate – not significant when compared with scramble control transfected cells (n = 4). (TIF) [file pone.0051111.s002.tif]

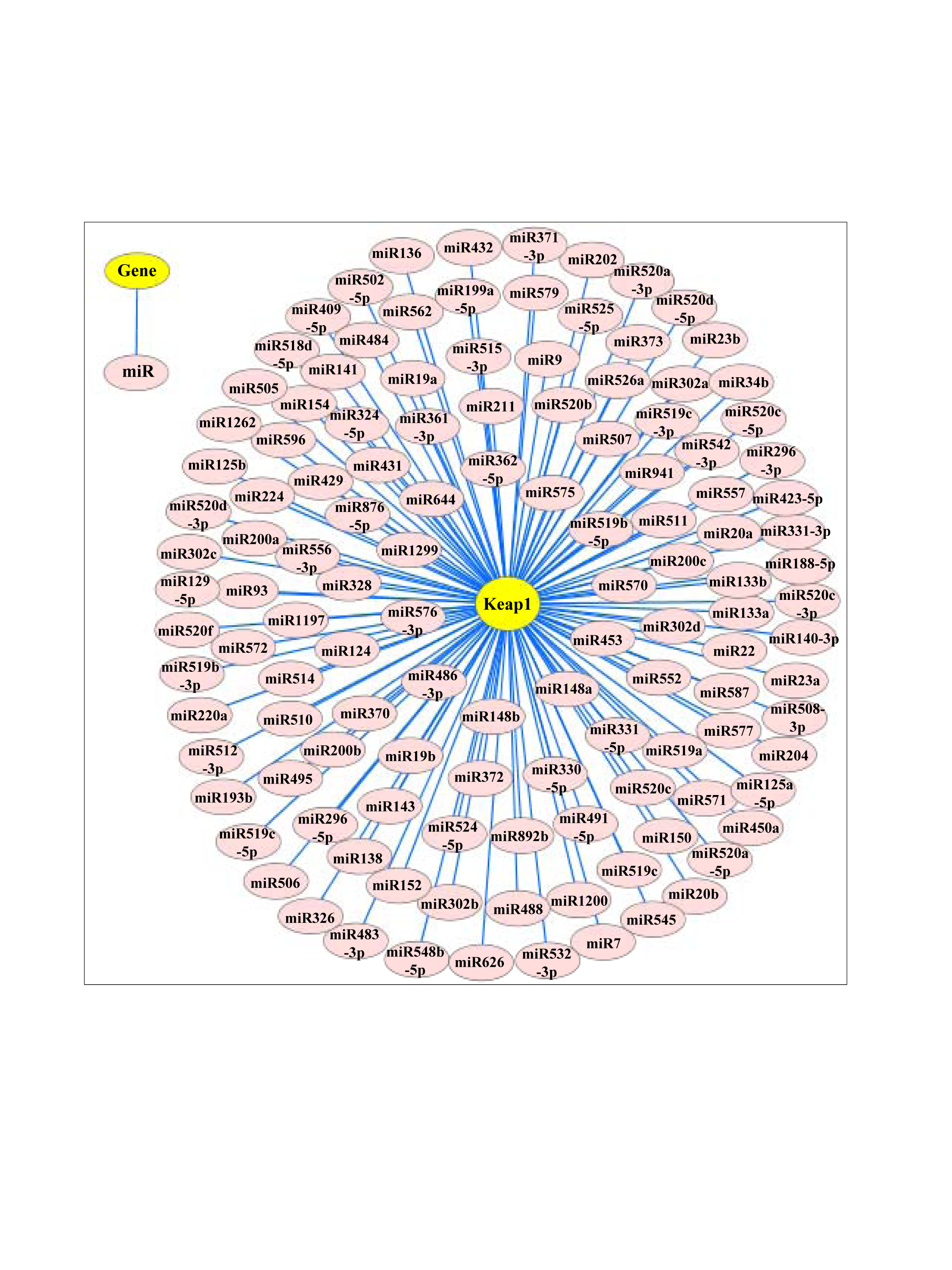

Supplement: Figure S3 — Schema of predicted Keap1 targeting miRs. Analysis of miRNAs that are predicted to target human Keap1 by “miRecords” and only the intersecting miR IDs from at least 2 individual databases are considered to generate Keap1:miR map using a network generating software “Cytoscape_v2.8.2”. (TIF) [file pone.0051111.s003.tif]

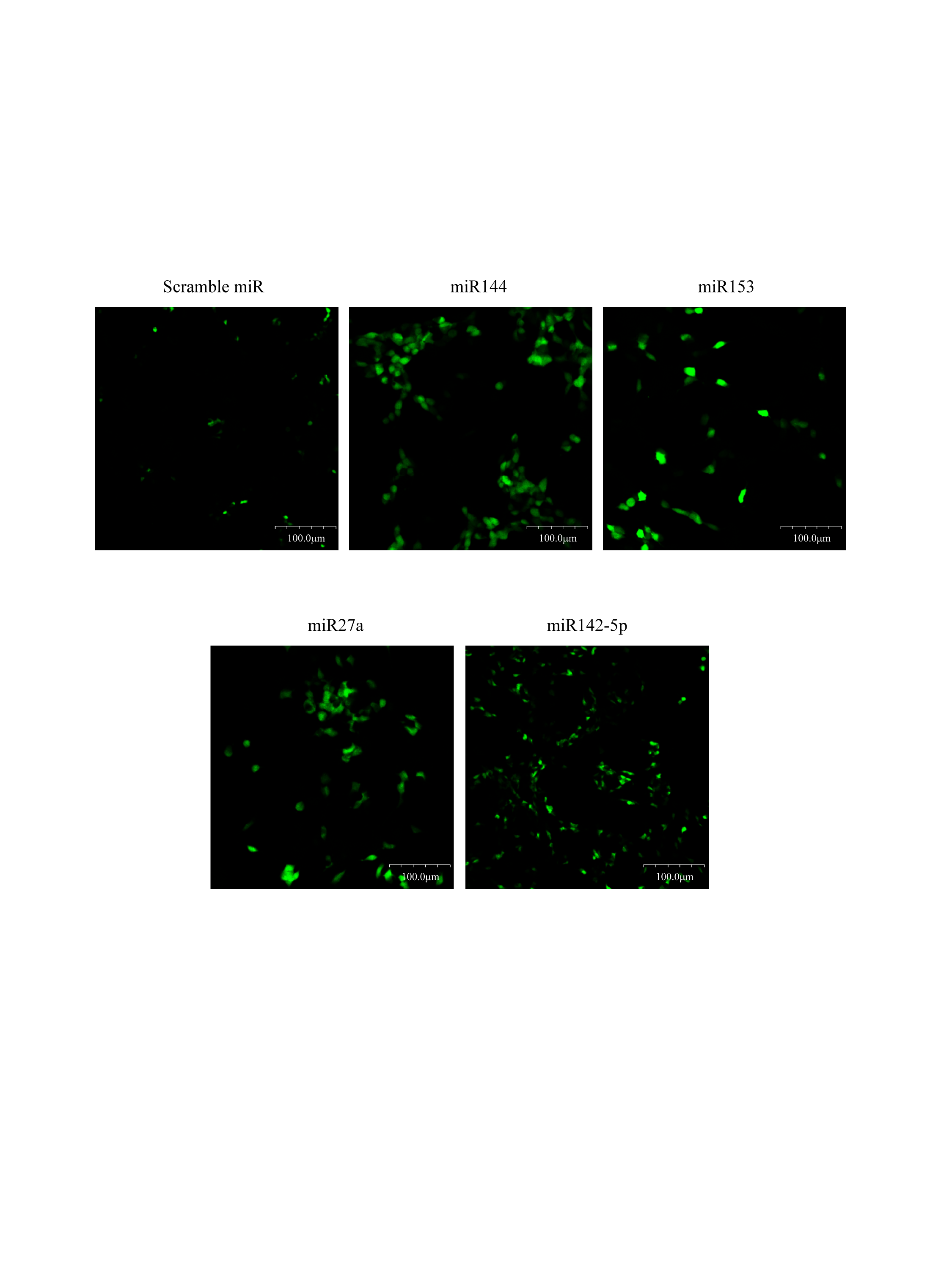

Supplement: Figure S4 — Effect of overexpression of different miRs on endogenous ROS levels. SH-SY5Y cells were transfected with either 100 nM of scramble miRNA or individual miRs for 48 h. At the end of the treatment, cells were treated with 5 µM of DCF-DA in dark and returned to incubator. After 30 min the cells were washed gently with PBS to remove excess fluorophore. Photomicrographs were generated using Olympus IX71 microscope. (TIF) [file pone.0051111.s004.tif]

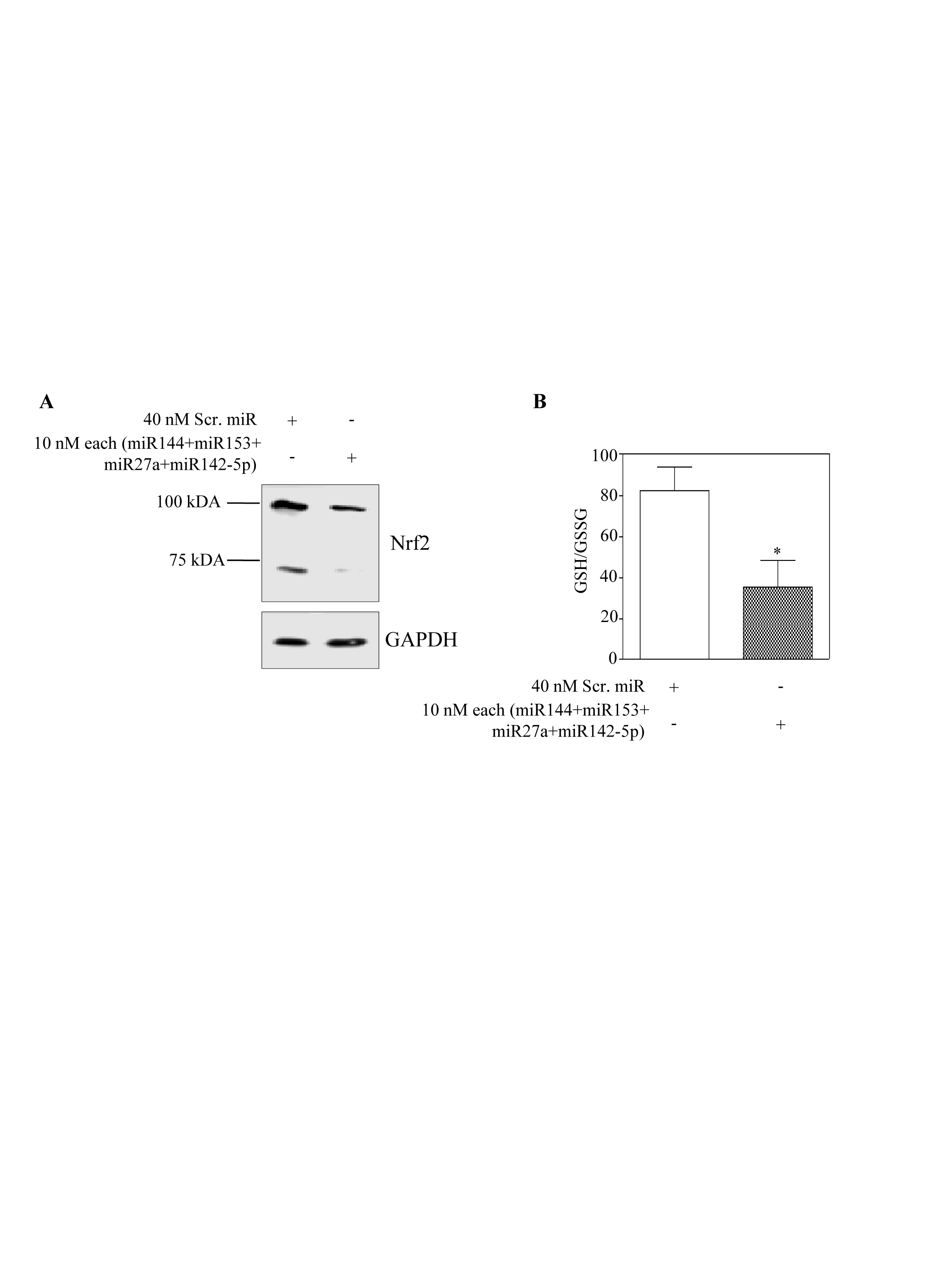

Supplement: Figure S5 — Effect of 10 nM each of combination of miR144, miR153, miR27a and miR142-5p on Nrf2 protein expression. SH-SY5Y cells were transfected with either 40 nM of scramble miRNA or combination of miRs (10 nM each of miR144, miR153, miR27a, miR142-5p) for 48 h. (A) Nrf2 immunoblotting was performed in whole cell protein lysates with anti-GAPDH serving as loading control. (B) As described in Materials and methods, GSH/GSSG Glo assay was performed using GSH as standard (Promega). GSH and GSSG levels were normalized to protein and results were expressed as GSH/GSSG ratio. * - indicate significant (p<0.05) and when compared with scramble control transfected cells (n = 4). (TIF) [file pone.0051111.s005.tif]

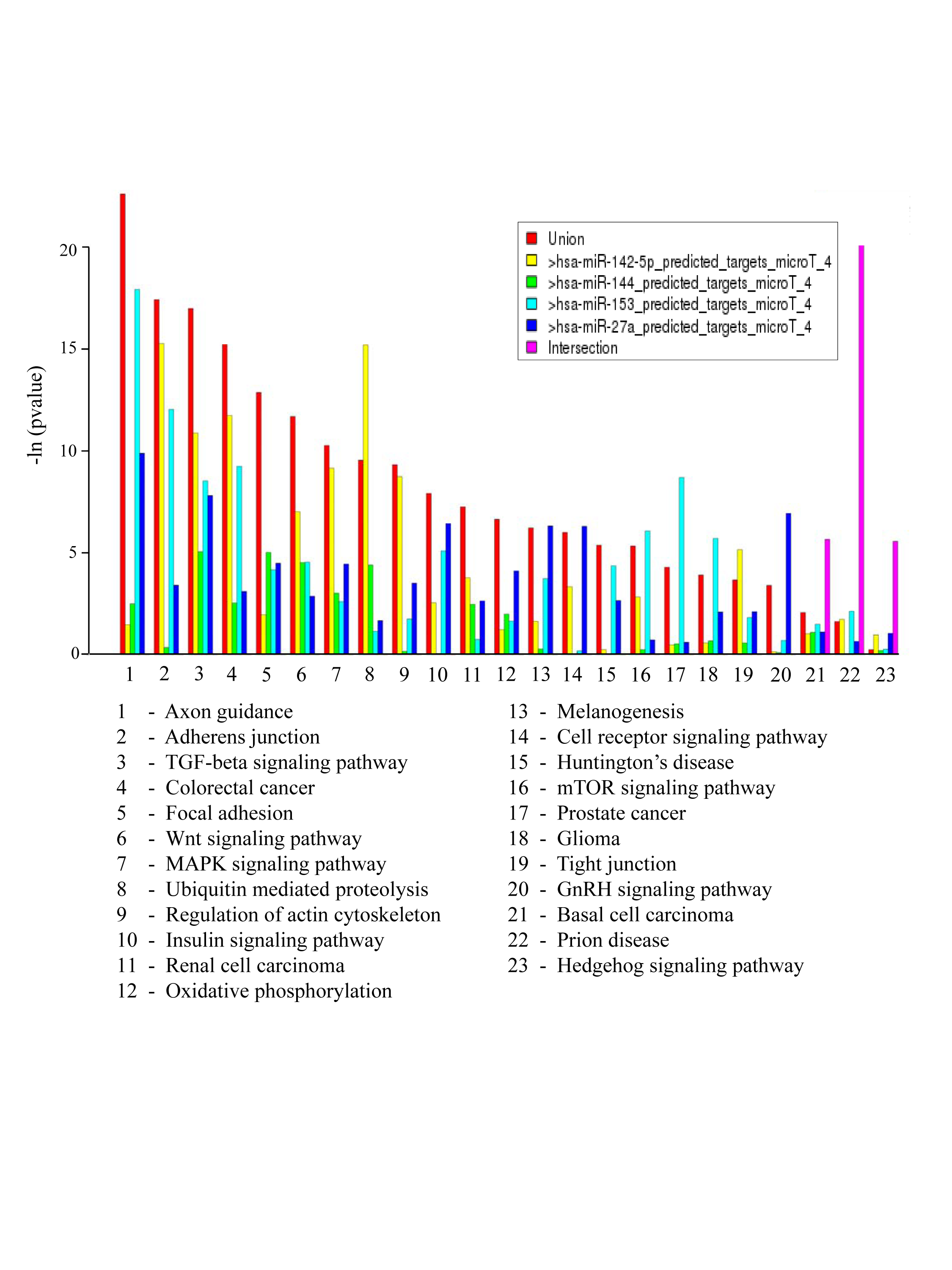

Supplement: Figure S6 — Integration analysis of multiple miRNAs (miR144/miR153/miR27a/miR142-5p) to various human pathways by DIANA mirPath. The effect of co-expression of this miRNA signature is visible in the bar plot graph of the –lnP values. In most of the top targeted pathways, the Union dataset –lnPs (Red bars) are higher than the –lnP values obtained for each single miRNA (Yellow, Green and Blue bars). (TIF) [file pone.0051111.s006.tif]
